# Distal Gastrectomy with Billroth II Reconstruction is Associated with Oralization of Gut Microbiome and Intestinal Inflammation: A Proof-of-Concept Study

**DOI:** 10.1245/s10434-020-08678-1

**Published:** 2020-06-05

**Authors:** Angela Horvath, Augustinas Bausys, Rasa Sabaliauskaite, Eugenijus Stratilatovas, Sonata Jarmalaite, Burkhard Schuetz, Philipp Stiegler, Rimantas Bausys, Vanessa Stadlbauer, Kestutis Strupas

**Affiliations:** 1 Department of Gastroenterology and Hepatology, Medical University of Graz, Graz, Austria; 2 Department of Abdominal Surgery and Oncology, National Cancer Institute, Vilnius, Lithuania; 3 Clinic of Gastroenterology, Nephrourology and Surgery, Institute of Clinical Medicine, Faculty of Medicine, Vilnius University, Vilnius, Lithuania; 4 National Cancer Institute, Vilnius, Lithuania; 5 Biovis Diagnostik, Limburg, Germany; 6 Department of Transplantation Surgery, Medical University of Graz, Graz, Austria

## Abstract

**Background:**

Subtotal gastrectomy with Billroth II reconstruction (SGB2) results in increased gastric pH and diminished gastric barrier. Increased gastric pH following PPI therapy has an impact on the gut microbiome, intestinal inflammation, and possibly patient health. If similar changes are present after SGB2, these can be relevant for patient health and long-term outcomes after surgery. The aim of the study is to investigate whether SGB2 is associated with specific changes in gut microbiome composition and intestinal inflammation.

**Patients and Methods:**

This cross-sectional proof-of-concept study includes patients after SGB2 (*n* = 14) for early gastric cancer and their nongastrectomized in-house relatives as controls (*n* = 8). Fecal microbiome composition, intestinal inflammation (fecal calprotectin), gut permeability (DAO, LBP, sCD14), systemic inflammation (CRP) markers, and gastrointestinal symptoms are investigated. This study is registered at ClinicalTrials.gov (NCT03418428).

**Results:**

Microbiome oralization following SGB2 was defined by an increase in *Escherichia*–*Shigella*, *Enterococcus*, *Streptococcus*, and other typical oral cavity bacteria (*Veillonella*, *Oribacterium*, and *Mogibacterium*) abundance. The fecal calprotectin was increased in the SGB2 group [100.9 (52.1; 292) vs. 25.8 (17; 66.5); *p* = 0.014], and calprotectin levels positively correlated with the abundance of *Streptococcus* (*r*_s_ = 0.639; *p*_adj_ = 0.023). Gastrointestinal symptoms in SGB2 patients were associated with distinct taxonomic changes of the gut microbiome.

**Conclusions:**

SGB2 is associated with oralization of the gut microbiome; intestinal inflammation and microbiome changes were associated with gastrointestinal symptoms. These novel findings may open gut microbiome as a new target for therapy to improve quality of life and general patient health in long-term survivors after SGB2.

Gastrectomy is the only potentially curative treatment option for gastric cancer (GC), one of the most common malignancies worldwide.^1^^,^^2^ Most patients with nonmetastatic GC require total or subtotal gastrectomy with extended lymph node dissection. The method to reconstruct the gastrointestinal (GI) tract integrity after subtotal gastrectomy (SG) remains controversial, while Billroth I (B1), Billroth II (B2), or Roux-en-Y (RY) are all available and acceptable methods.^3^ Irrespective of type of reconstruction, SG results in serious anatomical and physiological changes in the GI tract, including increase in gastric pH due to reduced secretion of the gastric acid.^4^^–^6 Therefore, there is a strong rationale to expect alterations that are typical for gastric acid suppression in the gut microbiome following SG.

Changes in the gastric and distal GI microbiome following gastric acid suppression have been proposed by studies investigating the impact of proton pump inhibitors (PPI) on the microbiome.^7^^–^10 PPI intake alters the composition and increases the diversity of the gastric microbiome.^7^ In the distal GI tract that is naturally rich in microbes, the microbial diversity decreases after PPI intake.^8^^–^10 Moreover, the fecal microbiome shows increased levels of predominantly oral bacteria, such as *Streptococcus, Veillonella, Rothia*, or *Oribacterium*, as well as an increase of potential pathogens, such as *Enterococcus, Escherichia*–*Shigella*, or *Haemophilus*, after PPI therapy. At the same time, autochtonous and beneficial bacteria, including *Faecalibacterium*, *Ruminococcaceae*, and *Lachnospiraceae*, decrease significantly.^8^^,^^9^^,^^11^^–^13 Recently, the increase in oral bacteria in the stool of patients with liver cirrhosis was linked to intestinal inflammation, gut barrier disruption, and 3-year mortality.^14^ The described alterations in the microbial composition were partly attributed to the loss of the gastric acid barrier.^15^ Since gastric pH increases after SG with B2 reconstruction (SGB2), we hypothesize that similar alterations of the microbiome might occur in gastrectomized patients. This study investigates whether SGB2 is associated with specific increased gastric pH-related changes in gut microbiome composition and intestinal inflammation.

## Patients and Methods

### Study Participants

Patients older than 18 years with a history of subtotal gastrectomy for early gastric cancer (EGC) were included in the study group (SGB2 group). The EGC was defined as invasive gastric cancer that invades no more deeply than the submucosa, irrespective of lymph node metastasis.^16^ EGC patients were selected to avoid the potential impact of the disease or intensive adjuvant chemotherapy on the gut microbiome. All patients underwent open subtotal gastrectomy with a D2 lymphadenectomy as described in the fourth version of the Japanese Gastric Cancer Treatment guidelines 17 at the National Cancer Institute, Vilnius, Lithuania. Following resection, the gastrointestinal tract integrity was reconstructed by the antecolic end to side gastrojejunostomy on a long loop with a handsewn anastomosis (Billroth II). Braun’s side to side jejunostomy was performed in all cases approximately 30 cm below gastrojejunostomy. Patient’s in-house relatives without a history of gastric surgery were included in the control group. The exclusion criteria for the participants were as follows: (1) chemotherapy or radiotherapy 12 months prior to inclusion, (2) gastric stump cancer, (3) usage of antibiotics, pro-, pre-, or synbiotics, H2-blocker, or PPI 1 month prior to inclusion, (4) history of any other gastrointestinal tract resections than SGB2, (5) recurrence of gastric cancer, and (6) current nongastric malignancies.

### Stool Sample Collection and Sequencing

To evaluate the gut microbiome, fresh stool samples were collected from the study participants and immediately stored at − 80 °C until the DNA extraction. DNA was extracted with the MagNA Pure LC DNA Isolation Kit III (Bacteria, Fungi) (Roche, Mannheim, Germany) according to the manufacturer’s instructions. Hypervariable region V1–V2 was amplified (primers: 27F-AGAGTTTGATCCTGGCTCAG; R357-CTGCTGCCTYCCGTA) and sequenced using Illumina Miseq technology (Illumina, Eindhoven, the Netherlands), as previously published.^18^ Sequencing data are made publicly available in the National Center for Biotechnology Information (NCBI) sequence read archive (accession no. PRJNA592441).

### Processing of Sequence Data

Raw sequencing data were processed using QIIME 2 tools on a local Galaxy instance (https://galaxy.medunigraz.at/).^19^ Denoising (primers removing, quality filtering, correcting errors in marginal sequences, removing chimeric sequences, removing singletons, joining paired-end reads, and dereplication) was done with DADA2.^20^ Taxonomy was assigned based on Silva 132 database release at 99% operational taxonomic unit level with a naïve Bayes classifier.

### Laboratory Assessment

Enzyme-linked immunsorbent assay (ELISA) was used to measure fecal calprotectin, serum diamine oxidase (DAO) (both: Immundiagnostik, Bensheim, Germany), lipopolysaccharide binding protein (LBP, Hycult Biotech, Uden, the Netherlands), and soluble CD14 (sCD14, R&D Systems, Minneapolis, MN).

### Gastrointestinal Symptoms

The Lithuanian versions of the European Organization for Research and Treatment (EORTC) Quality of Life Questionnaire Core 30 (QLQ-C30) and gastric cancer-specific module—EORTC QLQ-STO22—were used to assess patient’s quality of life. For the analysis, the answers to the questions on the abdominal discomfort, diarrhea, and abdominal bloating were dichotomized into “symptoms” and “no symptoms” categories. Gastrointestinal symptoms were associated with the microbiome composition.

### Statistical Analysis

For statistical analysis of microbiome compositions, the web-based application Calypso (version 8.84) was employed. Features were normalized by total sum scaling and square root transformation. Alpha diversity analysis was quantified by the Shannon index. For further analysis, features were summarized to the corresponding genera. Beta diversity was examined by principal coordinate analysis (PCoA) based on a Bray–Curtis dissimilarity matrix with analysis of similarity (ANOSIM), as well as redundancy analysis (RDA) with one or multiple explanatory variables. Analysis of composition of microbiomes (ANCOM) and linear discriminant analysis (LDA) effect size (LEfSe) were used to compare the abundance of genera between groups. Network analysis was used to visualize significant correlations between taxa in both the SGB2 and control groups using Spearman correlation and only considering positive associations.

For statistical analysis of patients’ characteristics and gut permeability data, SPSS 23 was used. Categorical variables were compared with the Chi squared test, and continuous variables with the Mann–Whitney *U* test. Spearman’s rank correlation coefficient was used to explore associations between variables. *p* value < 0.05 was considered to be significant. Benjamini–Hochberg correction was applied where appropriate.

## Results

**Fig. 1:**
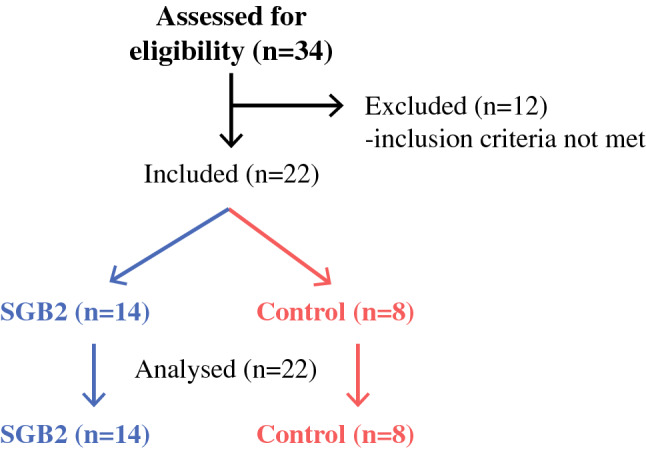
Trend flowchart of enrollment

Fourteen patients were included in the SGB2 group, and eight participants in the control group. The baseline clinicopathological characteristics are presented in Table 1. Six (75.0%) controls were spouses, and two (25.0%) were children. Participants in the control group were younger than the patients and predominantly female. The median time from surgery to enrollment was 45 (Q1;Q3: 26;63) months, while the minimum and maximum times were 6 and 101 months, respectively. Three (21.4%) patients received adjuvant chemotherapy following surgery, although in all cases, at least 12 months prior to the enrollment. None of the patients had disease recurrence at time of enrollment (Fig. 1).

**Table 1:** Clinicopathologic characteristics of study patients; data given as median (Q1; Q3)

	SGB2 (*n* = 14)	Controls (*n* = 8)	*p*
Age (years)	68 (64; 74)	59 (41; 65)	0.035
Sex			
Male	10 (71.4%)	1 (12.5%)	0.024
Female	4 (28.6%)	7 (87.5%)	
BMI (kg/m^2^)	24.7 (21.5; 28.4)	24.9 (22.0; 32.8)	0.616
Smoking	7 (50%)	1 (12.5%)	0.167
Systolic BP (mmHg)	130 (126; 135)	126 (122; 136)	0.525
Diastolic BP (mmHg)	79 (76; 90)	75 (71; 80)	0.297
CRP level (mg/l)	0.7 (0.3; 5.1)	0.8 (0.3; 1.6)	0.868
Tumor invasion			
Mucosal	5 (35.7%)	–	
Submucosal	9 (64.3%)	–	
Lymph node metastasis	4 (28.5%)		
Adjuvant chemotherapy	3 (21.4%)	–	
Medication			
Antihypertensive drugs (ACEI; BB; CCB; ARB, diuretics or combination)	10 (71.4%)	3 (37.5%)	0.187
Statins	1 (7.1%)	0 (0%)	0.999
Anticoagulants/antiplatelet drugs	2 (14.2%)	0 (0%)	0.515
Benzodiazepines/antipsychotic drugs/antidepressants	2 (14.2%)	1 (12.5%)	0.999
Gastrointestinal symptoms			
Abdominal discomfort	9 (69%)	5 (62.5%)	0.751
Diarrhea	7 (54%)	1(12.5%)	0.058
Bloating	6 (46%)	4 (50%)	0.864

*BMI* Body mass index, *BP* blood pressure, *CRP* C-reactive protein, *ACEI* angiotensin-converting enzyme inhibitors, *BB* beta-blockers, *CCB* calcium channel blockers, *ARB* angiotensin II receptor blockers

### Microbiome Composition

**Fig. 2:**
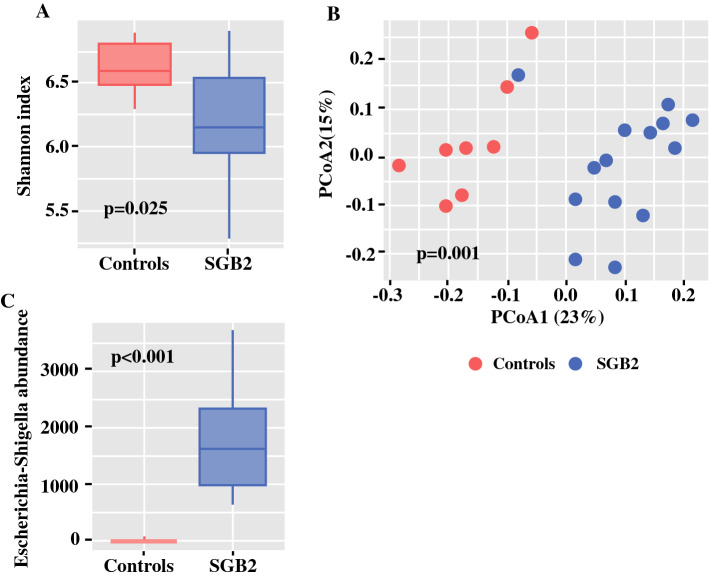
Changes in microbiome of SGB2 patients compared with controls: **a** Shannon index as a measurement of alpha diversity, **b** principal coordinate analysis plot based on Bray–Curtis dissimilarity, and **c** abundance of *Escherichia*–*Shigella* in microbiome of SGB2 patients and controls

In total, 2,042,502 sequencing reads were generated. After denoising, an average of 39,085 (min: 24,549; max: 49,361) reads per sample were available for analysis. Alpha diversity assessed by Shannon index after rarefication (sampling depth: 24,549 reads) was significantly decreased in gastrectomy patients compared with controls (*p* = 0.025, Fig. 2a). Median bacterial richness quantified by Chao1 index was comparable between groups (*p* = 0.69).

**Fig. 3:**
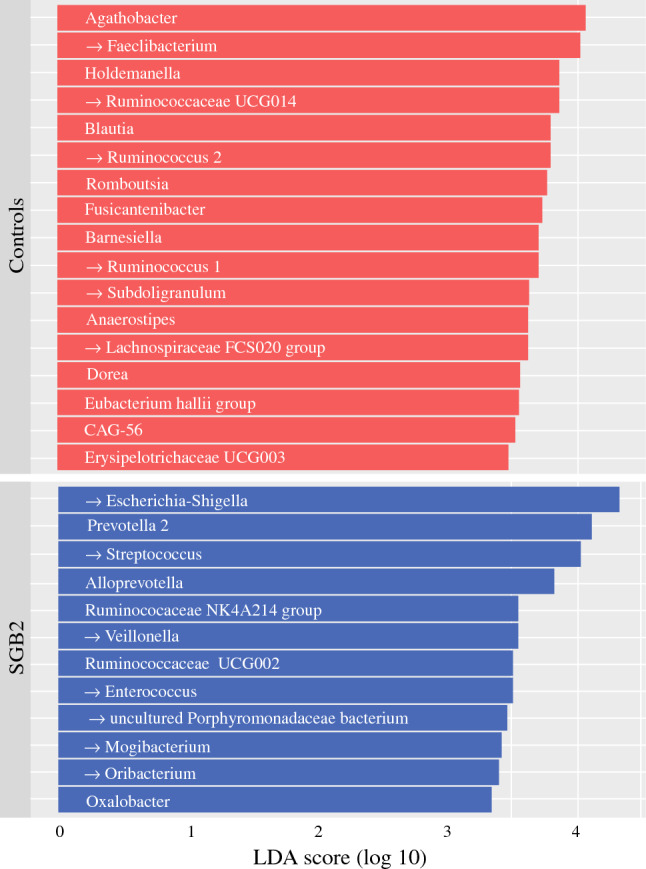
LDA effect size (LEfSe) results; genera marked with an arrow previously indicated in PPI-induced or PPI-associated dysbiosis

**Fig. 4:**
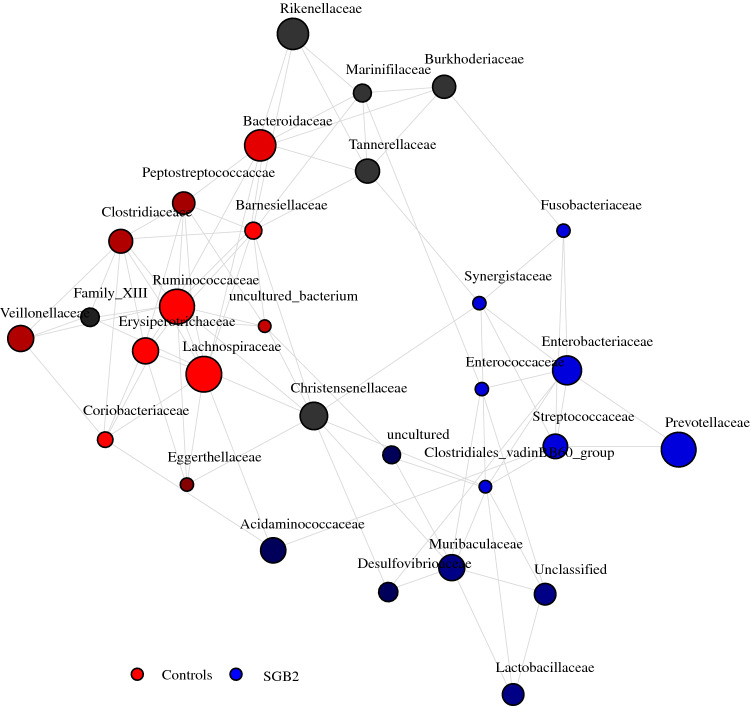
Network analysis representing positive correlations between 30 most abundant genera

Beta diversity analysis showed significant differences between the microbiome composition of patients and controls (ANOSIM: *r* = 0.442; *p* = 0.001) (Fig. 2b). ANCOM identified the genus *Escherichia*–*Shigella* to be more abundant in SGB2 patients compared with controls (fold-change = 302.7) (Fig. 2c). LEfSe corroborated this finding and attributed 11 additional genera to SGB2 and 17 genera to the control group. Of these 29 genera, 13 (45%) have been implicated in PPI-induced or PPI-associated dysbiosis in previous reports (Fig. 3). Network analysis of the 30 most abundant bacterial families showed associations between *Enterococcaceae*, *Synergistaceae, Enterobacteraceae, Fusobacteraceae, Streptococcaceae, Clostridiales* vadinBB60 group, and *Prevotellaceae* within the microbiomes of patients, and between *Barnesiellaceae, Bacteroidaceae, Ruminococcaceae, Lachnospiraceae, Erysipelotrichaceae*, and *Coriobacteriaceae* in the microbiomes of controls (Fig. 4). To exclude sex and age differences as potential confounders, RDA with multiple explanatory variables was performed but did not detect a significant influence of age (variance = 9.39; *F* = 1.07; *p* = 0.358) or sex (variance = 8.54; *F* = 0.97; *p* = 0.529) on the composition of the microbiome next to SGB2 (variance = 20.34; *F* = 2.32; *p* = 0.001).

### Inflammation and Gut Permeability

Fecal calprotectin as a marker of intestinal inflammation was significantly higher in SGB2 patients compared with controls. DAO, LBP, and sCD14 as markers for gut permeability and C-reactive protein (CRP) levels as a marker for systemic inflammation were comparable between groups. Details are given in Table 2.

**Table 2:** Intestinal inflammation and permeability markers in patients and controls; data given as median (Q1; Q3)

	SGB2 (*n* = 14)	Controls (*n* = 8)	*p*
Fecal calprotectin (ng/mg)	100.9 (52.1; 292)	25.8 (17; 66.5)	0.014
DAO (U/ml)	24.3 (11.2; 32.3)	19.6 (14.2; 27.2)	0.616
LBP (µg/ml)	15.8 (11.9; 20.1)	14.3 (11.6; 19.9)	0.868
sCD14 (µg/ml)	1.7 (1.6; 2.1)	1.7 (1.5; 1.9)	0.441

*DAO* Serum diamine oxidase, *LBP* lipopolysaccharide binding protein

Correlation analysis was done with all genera attributed either to the SGB2 or the control group (Fig. 3), and biomarkers of inflammation and gut barrier function. Fecal calprotectin was positively correlated with the abundance of *Streptococcus* (*r*_s_ = 0.639; *p*_adj_ = 0.023) and negatively correlated with the abundance of *Ruminococcaceae UCG014* (*r*_s_ = −0.755; *p*_adj_ = 0.002), *Barnesiella* (*r*_s_ = −0.748; *p*_adj_ = 0.002), *Ruminococcus 2* (*r*_s_ = −0.649; *p*_adj_ = 0.014), *Ruminococcus 1* (*r*_s_ = −0.616; *p*_adj_ = 0.022), and *Anaerostipes* (*r*_s_ = −0.572; *p*_adj_ = 0.041). Age, years since surgery, DAO, LBP, sCD14, and CRP levels were not significantly correlated with any of the indicated genera.

### Associations with Gastrointestinal Symptoms

**Fig. 5:**
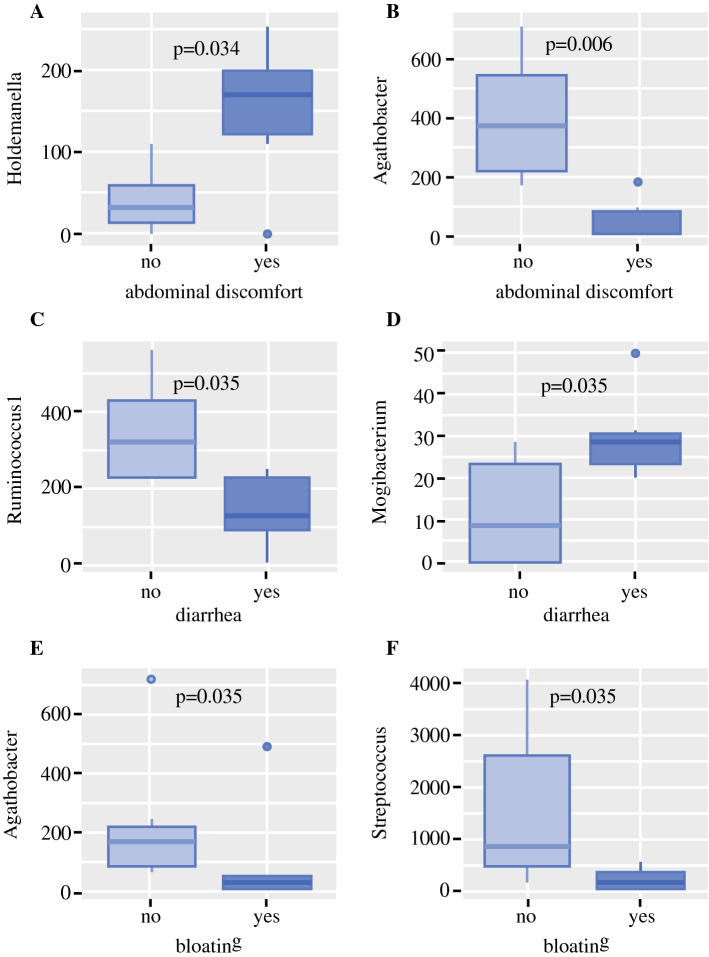
Symptom-related microbiome changes in patients after SGB2: **a**, **b** significant differences in genera of interest between patients with and without abdominal discomfort, **c**, **d** significant differences in genera of interest between patients with and without diarrhea, and **e**, **f** significant differences in genera of interest between patients with and without bloating

The most commonly documented gastrointestinal symptoms after SGB2 were abdominal discomfort (*n* = 9; 69%), diarrhea (*n* = 7; 54%), and bloating (*n* = 6; 46%). Patients who complained about abdominal discomfort showed higher abundance of *Holdemanella* (*p* = 0.034) and lower abundance of *Agathobacter* (*p* = 0.006) in their fecal microbiome. Diarrhea was associated with a significantly higher abundance of *Mogibacterium* (*p* = 0.035) and significantly lower abundance of *Ruminococcus 1* (*p* = 0.035). Patients who reported bloating showed a significantly lower abundance of *Agathobacter* (*p* = 0.035) and *Streptococcus* (*p *= 0.035). Details are shown in Fig. 5. Patients who suffered from diarrhea also showed significantly higher serum levels of CRP and a trend to higher calprotectin level in stool compared with patients without diarrhea [CRP (mg/l): 5 (0.4; 5.6) vs. 0.3 (0.3; 0.4), *p* = 0.035, respectively, and calprotectin (ng/mg): 371.4 (80.0; 526.5) vs. 66.2 (35.3; 100.9), *p* = 0.132, respectively]. DAO, LBP, and sCD14 levels were not different among patients with and without diarrhea. Neither abdominal discomfort nor bloating was associated with increased inflammation or gut permeability markers.

## Discussion

We investigated the alteration in the fecal microbiome of patients after SGB2. Our results clearly show the impact of SGB2 on the general gut microbiome composition, with decreased alpha diversity by Shannon index after SGB2 and significant differences in beta diversity between patients and healthy controls as well as taxonomic composition. Taxon comparisons revealed that approximately half of the genera with altered abundance have been linked to PPI therapy in previous studies. PPI intake increases the gastric pH from the physiological level of approximately 2.0 to over 6.0,^21^ considerably higher than pH 4, which is considered to be the threshold value for powerful bactericidal effect.^22^ Similar to PPI intake, SGB2 causes permanent increase of the gastric pH to values above 6.0.^23^ Therefore, our findings can be explained by the comparable loss of gastric barrier function after SGB2 and by PPI use. Vice versa, our results support the notion that PPI-induced microbiome changes are caused by acid suppression and are most likely not due to direct drug-induced effects on microbes.

The steep increase in *Escherichia*–*Shigella* was the most prominent difference between the microbiome of SGB2 patients and that of healthy controls. *Escherichia* is a common protagonist in small intestinal bacterial overgrowth (SIBO),^24^ which occurs in the majority of patients after gastrectomy and is associated with intestinal and postprandial symptoms.^25^ A similar observation was made in children after PPI therapy.^26^ Although members of the genus *Escherichia*–*Shigella* are not sensitive to pH variations in their environment, these seem to profit from the altered milieu, since these were also found to be increased in the general population after PPI intake.^8^^,^^27^ The observed increase in *Enterococcus*, a bacterium that is also often involved in SIBO, however, is directly attributable to the increased gastric pH. In a model of gastric barrier dysfunction, both genetic and pharmaceutical blockage of acid secretion in the stomach resulted in increased survival of orally gavaged *Enterococcus.*^15^ Moreover, after SGB2, patients showed a significant increase in *Streptococcus*. *Streptococcus* is a prevalent bacterial taxon in the oral cavity and the most commonly described bacterium in PPI-induced dysbiosis.^8^^,^^9^^,^^11^^–^13 This was recently linked to intestinal inflammation and gut permeability in cirrhosis patients.^14^ In the present study, we showed that *Streptococcus* is also associated with intestinal inflammation in patients after SGB2. Together with other oral bacteria (*Veillonella*, *Oribacterium*, and *Mogibacterium*), the observed increase in *Streptococcus* abundance supports the hypothesis of oralization after gastric acid barrier disruption, also in patients after SGB2. Furthermore, several beneficial commensals were decreased in the microbiome of SGB2 patients. The loss of these commensals correlated with the increase in calprotectin levels in stool. Especially the diminished abundance of *Faecalibacterium*, *Subdoligranulum*, and members of the *Ruminococcaceae* and *Lachnospiraceae* family again is similar to PPI dysbiosis.^9^^,^^11^^,^^12^^,^^14^

Besides the important pathophysiological information, our study may also have clinical implications for patients after SGB2. Chronic intestinal inflammation after SGB2 plays an important role in the patients’ health and quality of life. Although overall quality of life scores show an immediate deterioration after surgery followed by an increase to approximately normal levels within the first year, gastrointestinal symptoms remain a significant issue long after SG.^28^^–^30 In the present study, calprotectin levels were markedly increased in SGB2 patients and strongly associated with the presence of *Streptococcus* in the stool. A very similar pattern can be found in patients with long-term PPI use, in whom increased calprotectin levels and associations between oralization and inflammation have been described in previous reports.^14^^,^^31^^,^^32^ Chronic intestinal inflammation has been described in the pathogenesis of chronic diarrhea after SGB2.^33^ Intermittent or permanent chronic diarrhea is one of the most common problems in long-term survivors after gastrectomy,^28^^,^^34^^,^^35^ present in about 40% of patients.^36^ In the present study, approximately 54% of patients also suffered from diarrhea and showed higher calprotectin levels on average than patients without diarrhea, although this observation did not reach statistical significance, and validation in bigger studies is warranted. In patients with diarrhea, *Ruminococcus 1* was depleted, and *Mogibacterium* was overrepresented. *Ruminococcus 1* is a ubiquitous genus in the human microbiome that has the ability to degrade complex carbohydrates and provide nutrients for other commensals.^37^*Ruminococcus* species have been associated with a stable human microbiome in previous reports,^38^ and decreased abundance was associated with diarrhea in a porcine animal model.^39^*Mogibacterium* was found to be increased in Crohn’s disease and colorectal cancer patients.^40^^,^^41^ Other common gastrointestinal symptoms were abdominal discomfort and bloating. Both symptoms were associated with a decrease of *Agathobacter*. *Agathobacter* are butyrate producers who live in symbiosis with *Bifidobacteria*, giving them access to acetate as a substrate for butyrate production.^42^ Moreover, an increased abundance of *Holdemanella* was observed in patients with abdominal discomfort. Comprehensive studies on *Holdemanella* on human health are lacking, however, their taxonomic family *Erysipelotrichiaceae* contains highly immunogenic species and is associated with proinflammatory conditions.^43^ Interestingly, patients who reported bloating also showed a reduced abundance of *Streptococcus*. *Streptococcus* is the foremost genus in PPI-associated dysbiosis and has been linked to inflammation and gut barrier dysfunction before. However, the genus *Streptococcus* entails also beneficial species, such as *S. salivarius* subsp. *thermophilus* that is utilized in various probiotic products. VSL#3, which contains a *Streptococcus* species among others, has been shown to reduce bloating in patients with irritable bowel syndrome.^44^^,^^45^ Similarly, another multispecies probiotic containing *S. thermophilus* improved self-perceived gastrointestinal wellbeing.^46^ More in-depth studies are necessary to clarify the role of different *Streptococcus* species in gastrointestinal health and disease. Nevertheless, the associations between gastrointestinal symptoms and the microbiome in SGB2 patients highlight the importance of comprehensive studies in this field to improve patients’ postoperative outcomes and wellbeing.

Acid-unrelated changes in the microbiome of SGB2 patients include an increase of *Oxalobacter* abundance. *Oxalobacter* is an oxalate-metabolizing commensal that increases the colonic excretion of oxalate, which in turn, reduces the strain of calcium oxalate on the kidney.^47^ In the present study, *Oxalobacter* was exclusively found in patients after SGB2 and was absent in healthy controls. Although clinical trials that utilized *Oxalobacter* as a probiotic in patients with primary hyperoxaluria were unsuccessful,^48^ the natural occurrence of *Oxalobacter* after SGB2 might be a beneficial adaptation to the altered gastrointestinal physiology after SGB2.

The microbiome faces a variety of influencing factors, such as diet, gender, and age of the patient, that also need to be considered in cohort studies. By selecting in-house relatives as controls, we minimized the diet-related impact on gut microbiome composition as similar microbiome of individuals who share a household has already been shown previously,^49^^,^^50^ but we had to accept an age and gender bias. Our multivatriate analysis showed that the impact of age and gender was overshadowed by the strong influence of SGB2 on the microbiome composition. This was not unexpected since the age difference between the groups was rather small, and the changes in the microbiome after SGB2 such as the steep *Enterococcus* increase were more dominant compared with changes due to age. However, comparisons are hard to draw, since data on the aging microbiome are limited, and the findings are inconsistent.^51^^,^^52^ Gender-related differences in microbiome composition have been previously described in health and disease.^53^^–^55 Natural male predominance in the gastric cancer group and the expected female predominance in our control group might hinder the detection of gender-related differences further. Chemotherapy may also have an impact on gut microbiome composition. Dysbiosis has been described in the short term after chemotherapy application and linked to mucositis and impaired capability to resist pathogen colonization.^56^^,^^57^ However, there is a lack of data supporting whether dysbiosis persists in the long term, while this is still under investigation in an ongoing study.^58^ Chemotherapy may have some long-lasting slight impact on the gut microbiome composition, potentially similar to long-lasting imprint described in healthy adults after exposure to short-term broad-spectrum antibiotics.^59^ Therefore, in our present study, we could not rule out history of chemotherapy as a potential cofounder affecting microbiome, and excluded patients who received chemotherapy within the past 12 months.

Our results are in stark contrast to previously published sequencing data in patients with SG and B2 or RY reconstruction.^60^ In said study, the genera *Oxlobacter*, *Veillonella*, *Streptococcus*, *Escherichia*, *Shigella*, and *Oribacterium* among others were attributed to the control groups, while these were a crucial part of the microbiome alteration after SGB2 in the present study. Although the previous study had a rather big sample size, healthy controls were insufficiently characterized, and the use of medication was not analyzed as a potential confounder, which might lead to misinterpretation of the results. As we showed in our study, changes after gastrectomy can mimic drug-induced changes in the microbiome and, therefore, obscure the effect of the surgery. Especially, gastric pH-associated changes might be vulnerable to uncharacterized drug use in the control groups since PPI use is among the most dominant confounders in microbiome analysis in the general population.^61^ Large well-characterized cohorts are needed to fully elucidate this topic.

Our proof-of-concept study has several limitations. First is the relatively small sample size of the study. To prove the concept of increased gastric pH-related changes in the microbiome, the cross-sectional design of the present study was sufficient, although this is lacking data to show microbiome composition changes pre- and post-SGB2. Even with the relatively small but homogenous cohort and well-selected controls of this study, we were able to clearly confirm our hypothesis and show that SGB2 is associated with changes in the gut microbiome that can be attributed to the increased gastric pH. Second, our study investigated the fecal microbiome composition only in patients who underwent SG with B2 reconstruction. Therefore, it remains unclear whether other types of anastomosis, such as B1 or RY, might have the same impact on the gut microbiome. Future studies including all types of anastomosis will be important for generalization of our findings. However, since B1 gastroduodenal anastomosis is a common technique, especially in Asian countries,^62^ and RNY is the preferred method in Western countries,^63^ these studies might require prospective multicenter studies on an international scale. However, the same increase of gastric pH to the level above 6 has been reported after SG irrespective of B1 or B2 anastomosis; 23 therefore, it seems likely that the oralization of the gut microbiome phenomena would be attributable to the SG itself, irrespective of the reconstructive method.

## Conclusions

Our study shows that SGB2 is associated with microbiome oralization and intestinal inflammation. These findings prove that an increase in gastric pH irrespective of the reason for this increase is associated with typical microbiome changes. These novel findings may open gut microbiome as a new target for therapy to improve quality of life and general patient health in long-term survivors after SGB2.
